# Important Announcement

**Published:** 1890-01

**Authors:** F. E. Daniel

**Affiliations:** Editor


### IMPORTANT ANNOUNCEMENT.

The publication of Biography of Contemporary Physicians of Texas has been abandoned as a separate book. Failing to enlist sufficient pecuniary support, I clubbed the subscriptions with those of "Types of Successful Men of Texas," a work now in press by the Texas Biographical Publishing Company, and the data and portraits intended for the former have been turned over to Dr. Tobin and L. E. Daniell. The biography and picture of those of my subscribers who have given their consent to the consolidation, will be published in the work now in press; and all subscriptions that have been paid, or part paid, will be made good.

It is a handsome and very valuable book of some seven hundred pages, printed in clean, new style (Ronaldson) type, bound in pressed morrocco, gilt, and marbled edges. It contains, in addition to many of the most distinguished physicians of Texas, many prominent citizens, representatives of every class of society, in the professions, and in the various industries. For subscription and information, address Dr. J. J. Tobin, Austin, Texas. I still retain my connection with this company, and read and supervise all proof sheets. F. E. DANIEL, Editor.

